# Expression of members of the myf gene family in human rhabdomyosarcomas.

**DOI:** 10.1038/bjc.1991.461

**Published:** 1991-12

**Authors:** J. Clark, P. J. Rocques, T. Braun, E. Bober, H. H. Arnold, C. Fisher, C. Fletcher, K. Brown, B. A. Gusterson, R. L. Carter

**Affiliations:** Haddow Laboratories, Institute of Cancer Research, Sutton, Surrey, UK.

## Abstract

**Images:**


 © Macmillan Press Ltd., 1991

### Expression of members of the *myf* gene family in human rhabdomyosarcomas

J. Clark[1], P.J. Rocques[1], T. Braun[2], E. Bober[2], H.H. Arnold[2], C. Fisher[3], C. Fletcher[4], K. Brown[5], B.A. Gusterson[1], R.L. Carter[1] & C.S. Cooper[1]

[1]*Haddow Laboratories, Institute of Cancer Research, 15 Cotswold Road, Belmont, Sutton, Surrey SM2 5NG, UK;* [2]*University of Hamburg Medical School, Department of Toxicology, Grindelellee 117, D-2000 Hamburg 13, Germany;* [3]*Department of Histopathology, Royal Marsden Hospital, Fulham Road, London SW3 6JJ, UK; and* [4]*Department of Histopathology, St Thomas' Hospital, Lambeth Palace Road, London SE1, UK;* [5]*Department of Pathology and Microbiology, Medical School, University Walk, Bristol BS8 1TD, UK.*

**Summary** Northern analysis of tumour RNA has been used to examine the expression of members of the *myf* family of muscle determining genes (*myf*3, *myf*4, *myf*5 and *myf*6) in a series of 20 rhabdomyosarcomas. A 2.0 kb *myf*3 transcript was observed in 85% of tumours, a 1.8 kb *myf*4 transcript was detected in 70% of tumours and a 1.7 kb *myf*5 transcript was observed in 55% of tumours. Transcription of *myf*6 occurred in 28% of tumours, but there were several transcript sizes (1.2, 1.5, 2.0 and 3.5 kb) and in some individual tumours two or more transcripts were observed. Only two rhabdomyosarcomas, one classified as embryonal and one as pleomorphic, failed to exhibit transcription of members of the *myf* gene family. We were unable to detect transcription of *myf* genes in neuroblastomas, Wilms' tumours, hepatoblastomas, paediatric non-Hodgkin's lymphoma and leiomyosarcomas. When considered together these observations suggest that expression of *myf* genes could provide an extremely useful marker in the diagnosis of rhabdomyosarcoma.

The development of skeletal muscle is a complex process in which multipotent stem cells initially become committed to form mononuclear myoblasts. The myoblasts then fuse to form myotubes which in turn mature into striated muscle. Recent studies have demonstrated that the transition along this differentiation pathway may be determined, at least in part, by a family of trans-acting transcription factors that are directly involved in controlling the expression of muscle specific genes. The first genes found to encode muscle determining factors were the mouse MyoD1 gene (Davies *et al.*, 1987) and the rat myogenin gene (Wright *et al.*, 1989) which were both isolated by procedures involving subtractive cDNA hybridisation. Subsequently Braun *et al.* (1989*b*, 1990) isolated two related genes designated *myf*5 and *myf*6 from a human foetal muscle cDNA library and an additional two genes called *myf*3 and *myf*4 (Braun *et al.*, 1989*a*) that are the human homologues of, respectively, MyoD1 and myogenin. Each of the four *myf* genes encodes a highly conserved basic-helix-loop-helix region that is believed to be responsible for the binding of *myf* proteins to enhancer regions in muscle specific genes. In addition, each gene can convert mouse fibroblasts into cells with myogenic characteristics and in analyses of normal tissue all four genes were expressed exclusively in striated muscle (Braun *et al.*, 1989*a*,*b* 1990).

Rhabdomyosarcomas are tumours that show differentiation towards striated muscle. Conventionally, three main histological subtypes are recognised (Enzinger & Weiss, 1988). Most (70–80%) are embryonal rhabdomyosarcomas that frequently have the microscopic appearance of foetal muscle and vary in morphology from undifferentiated round cell tumours with few discernible myoblasts to well differentiated tumours containing a high proportion of myoblasts (Enzinger & Weiss, 1988). They usually arise in the first and early second decades of life and account for 6–7% of paediatric neoplasms. Alveolar rhabdomyosarcomas, which usually occur during the second and third decades, are composed of aggregates of poorly differentiated cells that are separated by bands of dense fibrous tissue. Finally, the rare pleomorphic rhabdomyosarcomas occur later in life and are characterised by the presence of haphazardly arranged bizarre cells. Diagnosis is often problematic particularly for poorly differentiated embryonal tumours which can be difficult to distinguish from other classes of paediatric round cell tumours, such as neuroblastoma, hepatoblastoma and non-Hodgkin's lymphoma (Enzinger & Weiss, 1988). The final tissue diagnosis frequently requires the use of supplementary techniques such as electron microscopy and, in particular, the use of immunohistochemical reagents which detect, for example, muscle-associated intermediate filaments, contractile proteins and myoglobin. The most commonly used antibodies are those directed against desmin, myoglobin, fast myosin and sarcomeric actin. Their interpretation is sometimes difficult particularly in poorly differentiated rhabdomyosarcomas where expression of muscle-associated proteins is limited (Schmidt *et al.*, 1988; Carter *et al.*, 1989; Dodd *et al.*, 1989; Carter *et al.*, 1990). In order to determine whether the muscle determining genes *myf*3, *myf*4, *myf*5 and *myf*6 can be used to assist in the diagnosis of rhabdomyosarcoma we have, in the present study, examined the expression of these genes in a series of rhabdomyosarcomas that included representatives from all three histological categories.

### Materials and methods

### *Tumours and cell lines*

Fresh specimens of primary soft tissue sarcomas were obtained from the Royal Marsden Hospital, London and Surrey, St Thomas' Hospital, London and The Hospital for Sick Children, Bristol, and stored at − 70°C. The cell lines RD, A204, A673 and Hs729 were obtained from the American Type Culture Collection and maintained under conditions recommended by the supplier. The RMS cell line (Garvin *et al.*, 1986) was kindly provided by Dr Julian Garvin.

### *Preparation of RNA*

Total cellular RNA was prepared from cell lines as described by Feramisco *et al.* (1982). To prepare RNA from tumour material the tumour (up to 0.1 g) was frozen in liquid nitrogen and ground into a powder with a pestle and mortar. The powder was added to 0.3 ml lysis solution (140 mm $NaCl_2$, 2 mM $MgCl_2$, 200 mM Tris-HCl, pH 8.5, 0.5%, v/v, Nonidet P40) containing 1.3 $\mu g\ ml^{-1}$ of the RNAase inhibitor Aluminon (Aldrich). The mixture was immediately vortexed for 5 s then centrifuged for 30 s at 8,000 *g*. The supernatant was recovered and mixed with 0.5 ml of phenol and 0.35 ml

Correspondence: C.S. Cooper.
Received 31 May 1991; and in revised form 4 July 1991.

of TSE (0.5%, w/v, SDS, 5 mM EDTA, 10 mM Tris HCl, pH 8.5) vortexed and subject to centrifugation for 1.5 min at 8,000 *g*. The aqueous phase was then extracted twice with 0.5 ml phenol and once with 0.5 ml chloroform. Finally, following the addition of 2.5 volumes of ethanol, the RNA was allowed to precipitate at − 20°C for 15 min, pelleted by centrifugation, redissolved in 40 µl of water and stored at − 20°C.

*Northern analysis*

Northern analysis was performed exactly as described previously (Stratton *et al.*, 1990) except that the hybridisation membrane was washed at 65°C with 1 × SSC containing 0.5% (w/v) SDS. The following cDNA hybridisation probes were used: a 0.8 kb *Eco*R1-*Eco*R1 *myf*3 fragment (Braun *et al.*, 1989*a*); a 1.3 kb *Eco*R1-*Eco*R1 *myf*4 fragment (Braun *et al.*, 1989*a*); a 1.1 kb *Bam*H1-*Bam*H1 *myf*5 fragment (Braun *et al.*, 1989*b*); a 1.2 kb *Eco*R1-*Eco*R1 *myf*6 fragment (Braun *et al.*, 1990); and a 1.1 kb PstI-PstI fragment of glyceraldehyde-3-phosphate dehydrogenase cDNA (kindly provided by Louise Howe).

### Results

*Expression of* myf *genes in human tumours*

Fresh tumours biopsies and human tumour cell lines have been examined for the expression of members of the *myf* gene family. The samples examined included 20 primary rhabdomyosarcomas, five rhabdomyosarcoma cell lines (RD, RMS, A204, Hs729, A673) and biopsies from representative classes of other paediatric tumours (neuroblastoma, Wilms' tumour, hepatoblastoma and non-Hodgkin's lymphoma) and of other soft tissue tumours (leiomyosarcomas). Fifteen rhabdomyosarcomas had an embryonal histology and were predominantly from children under 15. Of the remaining five rhabdomyosarcomas, three had an alveolar histology while there were single cases of pleomorphic and mixed embryonal/alveolar tumours.

To detect transcription of *myf* genes in primary rhabdomyosarcomas Northern blots of total cellular RNA were hybridised to $^{32}$P-labelled cDNA probes. In these experiments (Figure 1 and Table I) a 2.0 kb *myf*3 transcript was detected in 17/20 tumours, a 1.8 kb *myf*4 transcript was detected in 15/20 tumours, and a 1.7 kb *myf*5 transcript was found in 11/20 tumours. Transcription of *myf*6 was observed in 5/18 tumours but there were several different mRNA sizes (1.1, 1.5, 2.2 and 3.5 kb) and some tumours expressed more than one transcript. Thus STS259 contained both 1.5 and 3.5 kb transcripts while STS238 contained transcripts of 1.1, 1.5 and 2.2 kb (results not shown). Comparisons of the results obtained with the four *myf* probes failed to reveal consistent patterns of expression (Table I). Some rhabdomyosarcomas expressed all four *myf* genes. Other groups of tumours expressed (a) *myf*3, *myf*4 and *myf*5 but not *myf*6 (b) only *myf*3 and *myf*4 (c) only *myf*4 and *myf*5 and (d) only *myf*3 and *myf*5. Finally two tumours, one embryonal rhabdomyosarcoma (STS249), and one pleomorphic tumour (STS23) showed no evidence for transcription of *myf* genes.

Several rhabdomyosarcoma cell lines were also examined for expression of the four *myf* genes. RNA from two lines, RD and RMS contained abundant *myf*3 and *myf*4 transcripts but failed to hybridise to *myf*5 and *myf*6 probes (Figure 1). In contrast for the remaining three lines, A204,

**Figure 1** Expression of members of the *myf* gene family in human rhabdomyosarcomas and in the rhabdomyosarcoma cell lines RMS, Hs729, RD, A673 and A204 Northern blots of tumour RNA (10 µg per lane) were hybridised sequentially to *myf*3, *myf*4, *myf*5 and *myf*6 probes. The primary rhabdomyosarcomas examined are designated by their STS numbers (see Table I). Loading of RNA samples was assessed by staining RNA gels with ethidium bromide and by hybridisation of Northern blots to a glyceraldehyde-3-phosphate dehydrogenase (G3PD) cDNA probe.

**Table I** Expression of markers in human rhabdomyosarcomas

| | | | | | Expression of markers[b] | | | | | | | |
|---|---|---|---|---|---|---|---|---|---|---|---|---|
| *Tumour* | *Sex* | *Age* | *Site* | *Type*[a] | *myf*3 | *myf*4 | *myf*5 | *myf*6 | *Vimentin* | *Desmin* | *Fastmyosin* | *Myoglobin* |
| STS68 | F | 12 | Forearm | E | + | + | – | – | + | + | – | – |
| STS93 | M | 68 | Paratestis[c] | E | + | – | + | N.D. | + | + | + | + |
| STS172 | M | 16 | Testis[c] | E | + | + | + | + | + | + | + | + |
| STS235 | M | 18 | Perineum | E | + | + | – | – | + | + | – | – |
| STS237 | M | 8 | Retroperitoneum | E | + | – | – | – | + | + | – | + |
| STS238 | M | 15 | Paratestis | E | + | + | + | + | + | + | + | + |
| STS239 | F | 8 | Thigh | E | + | + | – | N.D. | + | + | + | + |
| STS240 | F | 11 | Forearm | E | + | + | – | – | + | + | – | – |
| STS246 | M | 11 | Paratestis | E | + | + | + | – | + | + | – | + |
| STS247 | M | 6 | Bladder | E | + | + | + | + | + | + | + | + |
| STS248 | M | 2 | Calf | E | + | + | + | – | + | + | + | + |
| STS249 | M | 5 | Paratestis | E | – | – | – | – | + | + | – | + |
| STS250 | M | 5 | Bladder | E | + | – | – | – | + | + | – | + |
| STS251 | M | 3 | Trunk | E | + | + | + | + | + | + | – | – |
| STS261 | F | 5 | Bladder | E | + | – | + | – | + | + | N.D. | N.D. |
| STS259 | F | 4 | Perianal | A | + | + | – | + | + | + | N.D. | – |
| STS69 | F | 12 | Perineum | A | + | + | + | – | + | + | N.D. | + |
| STS252 | M | 23 | Unknown | A | + | + | + | – | + | + | + | + |
| STS253 | M | 27 | Neck | A/E | – | + | + | – | + | + | N.D. | – |
| STS23 | M | 57 | Buttock | P | – | – | – | – | + | + | N.D. | + |

[a]Embryonal (E), alveolar (A), or pleomorphic (P); [b]Expression of *myf*3, *myf*4, *myf*5 and *myf*6 were determined by Northern analysis as described in the Materials and methods and illustrated in Figure 1. The presence of vimentin, desmin, fastmyosin and myoglobin were determined using antibodies as described by Carter *et al.* (1989, 1990); [c]These samples were taken from metastatic lymph node tumours.

A673 and Hs729 we found no evidence for *myf* gene expression. An interesting correlation was observed between the presence of *myf* gene transcripts and cell morphology. Thus the two cell lines (RD and RMS) expressing *myf*3 and *myf*4 contained a significant proportion of spindle shaped cells that had the appearance of myoblasts while the lines that did not express members of the *myf* gene family had an undifferentiated appearance (results not shown). Similar results were obtained by Hiti *et al.* (1989) who detected MyoD1 (*myf*3) in RD cells, but not in A204 and A673 cells, and noticed the same correlation between MyoD1 expression and cell morphology.

To determine whether *myf* genes are expressed in other classes of paediatric tumours we have examined three Wilms' tumours, two neuroblastomas, two hepatoblastomas, and three non-Hodgkin's lymphomas and, as an additional control, we analysed two smooth muscle tumours (leiomyosarcomas). Transcription of *myf* genes was not detected in these tumours (results not shown).

Since each member of the *myf* gene family contains a short conserved basic-helix-loop-helix region (Braun *et al.*, 1989*a*, 1990), the possibility arose that particular *myf* gene probes might cross hybridise to transcripts from other family members. We believe that this is unlikely since, as described above, comparisons of the levels of transcripts observed with each of the four *myf* gene probes revealed many distinct patterns of hybridisation. In addition, the sizes of several of the major *myf*6 transcripts (1.2, 1.4 and 3.5 kb) were quite distinct from those observed for *myf*3, *myf*4 and *myf*5 (1.7–2.0 kb). It could also be suggested that the signal resulted from contamination with normal striated muscle. Again we believe that this is unlikely because (a) care was taken to remove normal tissue before the samples were stored (b) many of the tumours were from sites which did not contain striated muscle and (c) when compared on the same Northern blot the signal observed for tumour RNA was usually much more intense than that observed for RNA from striated muscle (result not shown).

There is some evidence that the expression of *myf*5 is correlated with the stage of muscle differentiation. Thus levels of *myf*5 transcripts were high in early foetal skeletal muscle but dropped considerably in adult muscle (Braun *et al.*, 1989*a*,*b*). We were therefore interested to see whether the level of expression of the *myf* genes correlated with the degree of differentiation of rhabdomyosarcomas, which can be assessed by examining the immunophenotype defined by antibodies that detect muscle associated epitopes such as desmin, fast myosin and myoglobin (Carter *et al.*, 1990). Myoglobin and fast myosin are usually associated with well differentiated elements that often reveal morphological features of differentiation towards striated muscle in conventionally stained sections. By comparison desmin is expressed in a broader spectrum of rhabdomyosarcomas. Unfortunately for the present fairly small groups of rhabdomyosarcomas we failed to find any correlation between *myf* gene expression and degree of differentiation as determined by immunohistochemical analysis (Table I).

### Discussion

Northern analysis of tumour RNA has been used to demonstrate that the *myf*3 gene is expressed in a high proportion of primary rhabdomyosarcomas. These results are in agreement with studies carried out in other laboratories. Using a mouse MyoD1 cDNA probe Hiti *et al.* (1989) detected transcripts in four out of five primary embryonal rhabdomyosarcomas, and two out of three rhabdomyosarcomas growing as explants *in vitro*. Similarly in studies on fresh rhabdomyosarcomas Scrable *et al.* (1989) detected MyoD1-related transcripts in five out of five alveolar tumours and eight out of eight embryonal tumours. We have now extended these analyses to other members of the *myf* gene family. Our results show that *myf*3 was expressed in the majority of rhabdomyosarcomas (17/20) usually together with *myf*4. By comparison the *myf*5 and *myf*6 genes, although yielding abundant transcripts in some rhabdomyosarcomas, were expressed in a lower proportion of tumours: 11/20 for *myf*5 and 6/18 for *myf*6.

Members of the *myf* gene family are apparently expressed quite infrequently in other classes of tumour. Both Hiti *et al.* (1989) and Scrable *et al.* (1989) failed to detect MyoD1-related transcripts in other groups of paediatric tumours and in soft tissue tumours. Furthermore, in the present study we failed to detect transcription of the four *myf* genes in Wilms' tumour, neuroblastoma, hepatoblastoma, paediatric non-Hodgkin's lymphoma and leiomysarcoma. Since *myf* gene expression appears to be restricted to rhabdomyosarcoma, it is possible that the expression of these genes may prove useful in the diagnosis of rhabdomyosarcoma. In this regard *myf*3 and *myf*4, which are both expressed in a high proportion of rhabdomyosarcomas may be particularly useful.

One embryonal tumour (STS249) and one pleomorphic tumour (STS23) failed to show *myf* gene expression. However both tumours expressed desmin and myoglobin (Table I) and the diagnoses were considered to be sound. It is conceivable that the absence of *myf* gene expression is simply a reflection of the insensitivity of Northern analysis when compared, for example, to the immunohistochemical methods that were used to detect desmin and myoglobin. A major advantage of immunohistochemical methods is that they can be used to detect expression of proteins in small pieces of tumour. Indeed, if expression of *myf* genes is to become widely accepted as a marker in the diagnosis of rhabdomyosarcomas, it will be necessary to produce antibodies for use in routine immunohistochemical studies which ideally could be used to examine formalin fixed tissue.

In conclusion we have demonstrated that each member of the gene family *myf*3, *myf*4, *myf*5 and *myf*6, is expressed in rhabdomyosarcomas. In addition, since the great majority of rhabdomyosarcomas express one or more of these genes and their expression was not detected in other classes of paediatric tumour, they could prove extremely useful in the diagnosis of rhabdomyosarcomas. However, if this method is to be adapted for routine use in histopathology laboratories, antibodies that recognise the *myf* proteins will be required. Indeed it is probable that the production of these antibodies should represent a major objective of future studies.

This work was funded by grants from the Cancer Research Campaign and Medical Research Council. We would like to thank Christine Bell for typing this manuscript and Jem Berry for supplying some of the tumour tissue. K.B. is a CLIC Senior Research Fellow.

### References

BRAUN, T., BOBER, E., BUSCHHAUSEN-DENKER, G., KOHTZ, S., GRZESCHIK, K.-H. & ARNOLD, H.H. (1989*a*). Differential expression of myogenic determining genes in muscle cells: possible autoactivation by the *myf* gene products. *EMBO J.*, **8**, 3617.

BRAUN, T., BUSCHHAUSEN-DENKER, G., BOBER, E., TANNICH, E. & ARNOLD, H.H. (1989*b*). A novel human muscle factor related to but distinct from MyoD1 induces myogenic conversion in 10T1/2 fibroblasts. *EMBO J.*, **8**, 701.

BRAUN, T., BOBER, E., WINTER, B., ROSENTHAL, N. & ARNOLD, H.H. (1990). *Myf*6, a new member of the human gene family of myogenic determination factors: evidence for a gene cluster on chromosome 12. *EMBO J.*, **9**, 821.

CARTER, R.L., MCCARTHY, K.P., MACHIN, L.G., JAMESON, C.F., PHILP, E.R. & PINKERTON, C.R. (1989). Expression of desmin and myoglobin in rhabdomyosarcomas and in developing skeletal muscle. *Histopathology*, **15**, 585.

CARTER, R.L., JAMESON, C.F., PHILP, E.R. & PINKERTON, C.R. (1990). Comparative phenotypes in rhabdomyosarcoma and developing skeletal muscle. *Histopathology*, **17**, 301.

DAVIS, R.L., WEINTRAUB, H. & LASSER, A.B. (1987). Expression of a single transfected cDNA converts fibroblasts into myoblasts. *Cell*, **51**, 987.

DODD, S., MALONE, M. & MCCULLOCH, W. (1989). Rhabdomyosarcoma in children: a histological and immunological study of 59 cases. *J. Pathol.*, **158**, 13.

ENZINGER, F.M. & WEISS, S.W. (1988). *Soft Tissue Tumours*. The C.V. Mosby Company.

FERAMISCO, J.R., SMART, J.E., BURRIDGE, K., HELFMAN, D.M. & THOMAS, G.P. (1982). Co-existence of vinculin and a vinculin-like protein of higher molecular weight in smooth muscle. *J. Biol. Chem.*, **257**, 11024.

GARVIN, J.A., STANLEY, W.S., BENNETT, D.D., SULLIVAN, J.L. & SENS, D.A. (1986). The *in vitro* growth, heterotransplantation and differentiation of a human rhabdomyosarcoma cell line. *Am. J. Pathol.*, **125**, 208.

HITI, A.L., BOGENMANN, E., CONZALES, F. & JONES, P.A. (1989). Expression of the MyoD1 muscle determining gene defines differentiation capability but not tumorgenecity of human rhabdomyosarcomas. *Mol. Cell. Biol.*, **9**, 4722.

SCHMIDT, R.A., CANE, R., HAAS, J.E. & GOWN, A.M. (1988). Diagnosis of rhabdomyosarcoma with HHF35, a monoclonal antibody directed against muscle actins. *Am. J. Pathol.*, **131**, 19.

SCRABLE, H., WHITTE, D., SHIMADA, H. & 7 others (1989). Molecular differential pathology of rhabdomyosarcoma. *Genes. Chr. Cancer*, **1**, 23.

STRATTON, M.R., MOSS, S., WARREN, W. & 8 others (1990). Mutation of the p53 gene in human soft tissue sarcomas; association with abnormalities of the RB1 gene. *Oncogene*, **5**, 1297.

WRIGHT, W.E., SASSOON, D.A. & LIN, V.J. (1989). Myogenin, a factor regulating myogenesis has a domain homologous to myoD. *Cell*, **56**, 607.


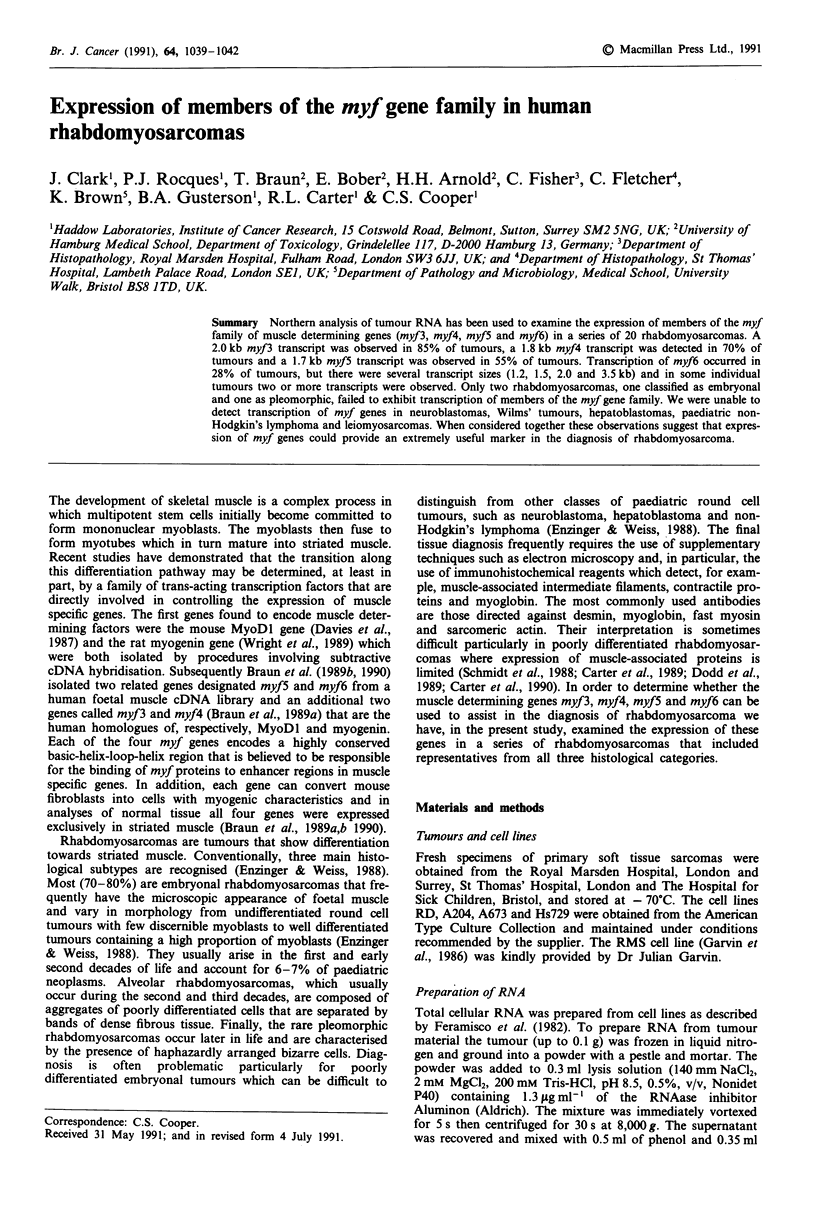

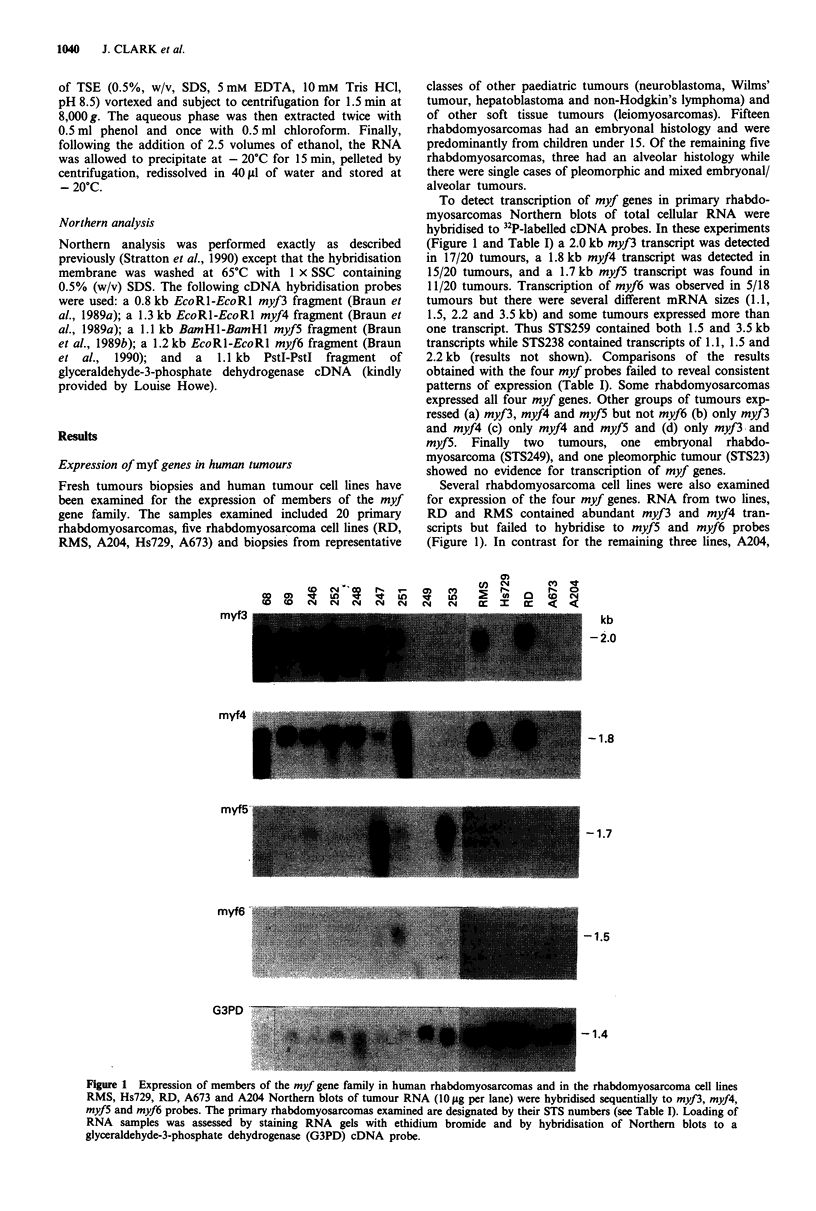

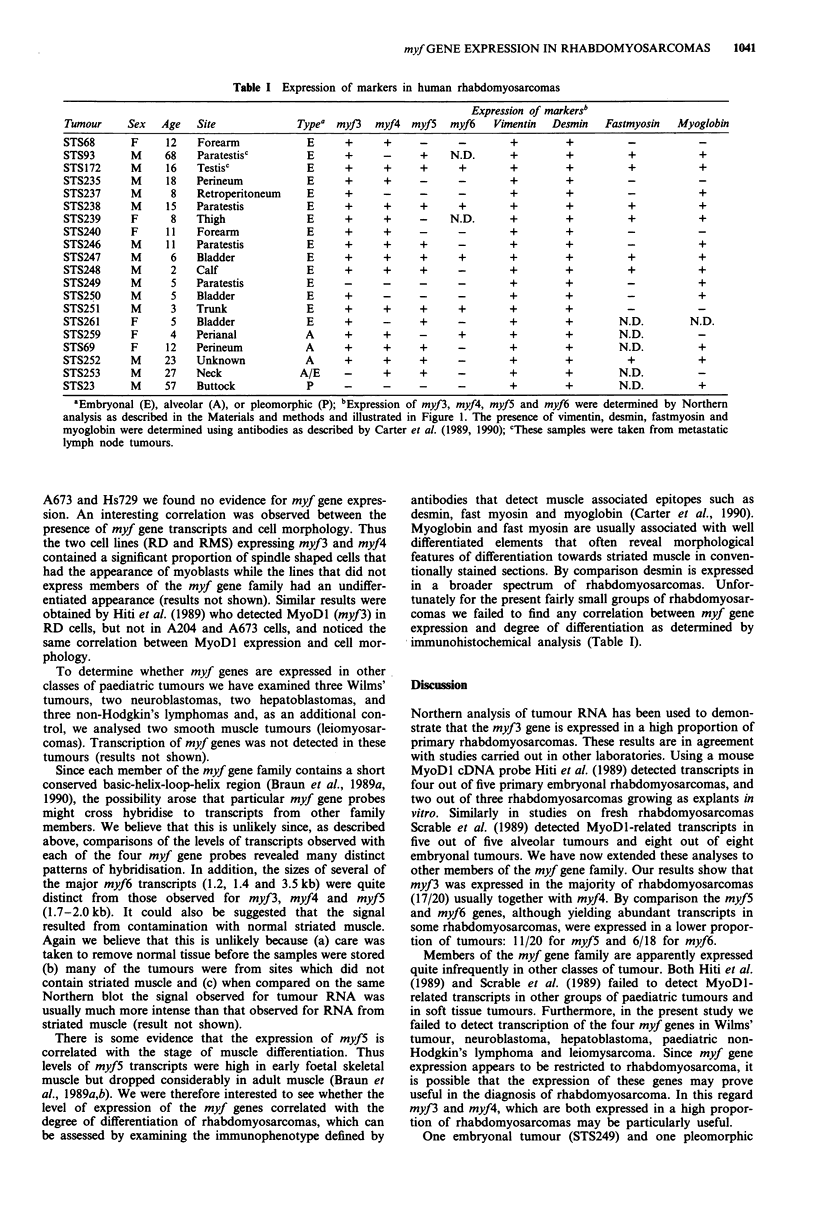

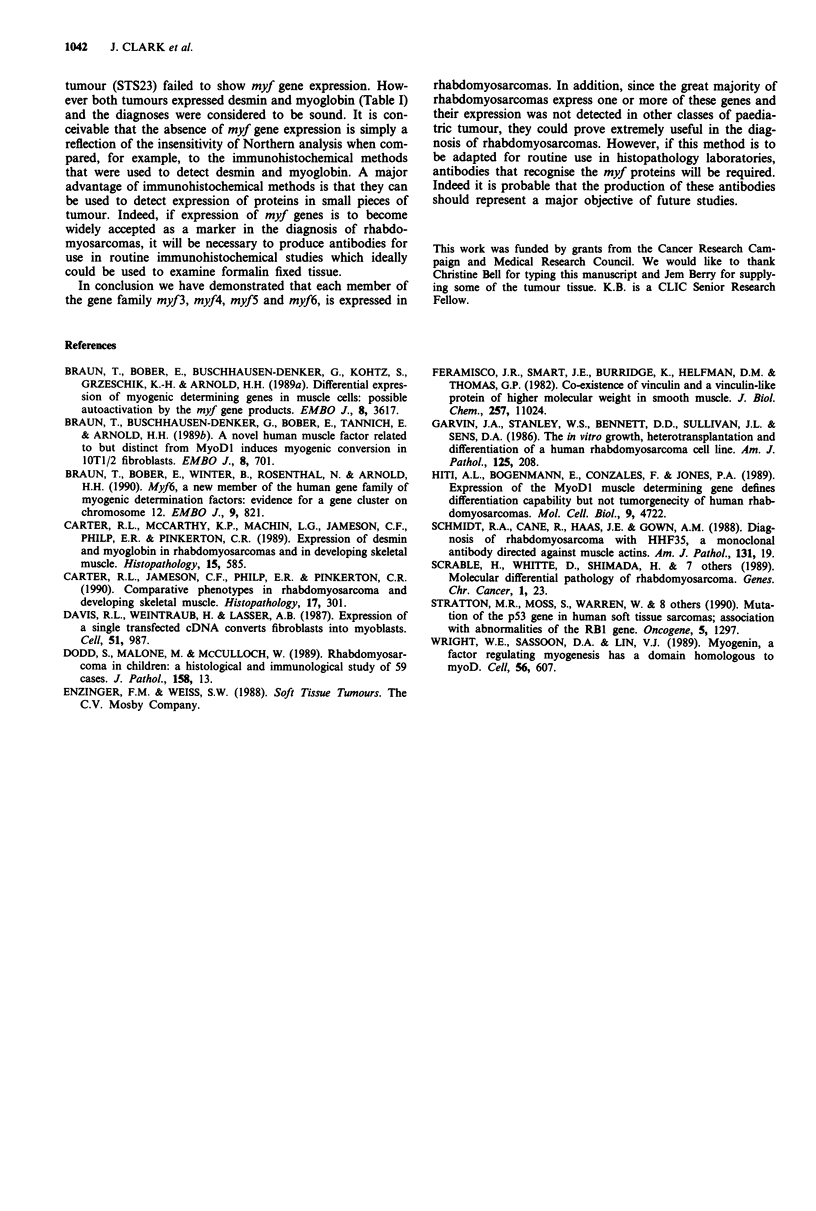


## References

[OCR_00410] Braun T., Bober E., Buschhausen-Denker G., Kohtz S., Grzeschik K. H., Arnold H. H., Kotz S. (1989). Differential expression of myogenic determination genes in muscle cells: possible autoactivation by the Myf gene products.. EMBO J.

[OCR_00422] Braun T., Bober E., Winter B., Rosenthal N., Arnold H. H. (1990). Myf-6, a new member of the human gene family of myogenic determination factors: evidence for a gene cluster on chromosome 12.. EMBO J.

[OCR_00416] Braun T., Buschhausen-Denker G., Bober E., Tannich E., Arnold H. H. (1989). A novel human muscle factor related to but distinct from MyoD1 induces myogenic conversion in 10T1/2 fibroblasts.. EMBO J.

[OCR_00434] Carter R. L., Jameson C. F., Philp E. R., Pinkerton C. R. (1990). Comparative phenotypes in rhabdomyosarcomas and developing skeletal muscle.. Histopathology.

[OCR_00428] Carter R. L., McCarthy K. P., Machin L. G., Jameson C. F., Philp E. R., Pinkerton C. R. (1989). Expression of desmin and myoglobin in rhabdomyosarcomas and in developing skeletal muscle.. Histopathology.

[OCR_00439] Davis R. L., Weintraub H., Lassar A. B. (1987). Expression of a single transfected cDNA converts fibroblasts to myoblasts.. Cell.

[OCR_00444] Dodd S., Malone M., McCulloch W. (1989). Rhabdomyosarcoma in children: a histological and immunohistochemical study of 59 cases.. J Pathol.

[OCR_00453] Feramisco J. R., Smart J. E., Burridge K., Helfman D. M., Thomas G. P. (1982). Co-existence of vinculin and a vinculin-like protein of higher molecular weight in smooth muscle.. J Biol Chem.

[OCR_00459] Garvin A. J., Stanley W. S., Bennett D. D., Sullivan J. L., Sens D. A. (1986). The in vitro growth, heterotransplantation, and differentiation of a human rhabdomyosarcoma cell line.. Am J Pathol.

[OCR_00465] Hiti A. L., Bogenmann E., Gonzales F., Jones P. A. (1989). Expression of the MyoD1 muscle determination gene defines differentiation capability but not tumorigenicity of human rhabdomyosarcomas.. Mol Cell Biol.

[OCR_00471] Schmidt R. A., Cone R., Haas J. E., Gown A. M. (1988). Diagnosis of rhabdomyosarcomas with HHF35, a monoclonal antibody directed against muscle actins.. Am J Pathol.

[OCR_00480] Stratton M. R., Moss S., Warren W., Patterson H., Clark J., Fisher C., Fletcher C. D., Ball A., Thomas M., Gusterson B. A. (1990). Mutation of the p53 gene in human soft tissue sarcomas: association with abnormalities of the RB1 gene.. Oncogene.

[OCR_00485] Wright W. E., Sassoon D. A., Lin V. K. (1989). Myogenin, a factor regulating myogenesis, has a domain homologous to MyoD.. Cell.

